# MoReLab: A Software for User-Assisted 3D Reconstruction

**DOI:** 10.3390/s23146456

**Published:** 2023-07-17

**Authors:** Arslan Siddique, Francesco Banterle, Massimiliano Corsini, Paolo Cignoni, Daniel Sommerville, Chris Joffe

**Affiliations:** 1 Department of Computer Science, Pisa University, Largo Bruno Pontecorvo 3, 56127 Pisa, Italy; arslan.siddique@isti.cnr.it; 2 Visual Computing Laboratory, ISTI-CNR, Via G. Moruzzi 1, 56124 Pisa, Italy; massimiliano.corsini@isti.cnr.it (M.C.); paolo.cignoni@isti.cnr.it (P.C.); 3 EPRI, 3420 Hillview Avenue, Palo Alto, CA 94304, USA; dsommerville@epri.com (D.S.); cjoffe@epri.com (C.J.)

**Keywords:** image-based 3D reconstruction, 3D modeling, user-assisted 3D reconstruction, video-based 3D reconstruction, Structure from Motion, HCI

## Abstract

We present MoReLab, a tool for user-assisted 3D reconstruction. This reconstruction requires an understanding of the shapes of the desired objects. Our experiments demonstrate that existing Structure from Motion (SfM) software packages fail to estimate accurate 3D models in low-quality videos due to several issues such as low resolution, featureless surfaces, low lighting, etc. In such scenarios, which are common for industrial utility companies, user assistance becomes necessary to create reliable 3D models. In our system, the user first needs to add features and correspondences manually on multiple video frames. Then, classic camera calibration and bundle adjustment are applied. At this point, MoReLab provides several primitive shape tools such as rectangles, cylinders, curved cylinders, etc., to model different parts of the scene and export 3D meshes. These shapes are essential for modeling industrial equipment whose videos are typically captured by utility companies with old video cameras (low resolution, compression artifacts, etc.) and in disadvantageous lighting conditions (low lighting, torchlight attached to the video camera, etc.). We evaluate our tool on real industrial case scenarios and compare it against existing approaches. Visual comparisons and quantitative results show that MoReLab achieves superior results with regard to other user-interactive 3D modeling tools.

## 1. Introduction

Three-dimensional (3D) reconstruction is the process of creating a three-dimensional representation of a physical object or environment from two-dimensional images or other sources of data. The goal of 3D reconstruction is to create a digital model that accurately represents the shape, size, and texture of the object or environment. It can create accurate models of buildings, terrain, and archaeological sites, as well as virtual environments for video games and other applications. These 3D models can be created by automatic scanning of static objects using LiDAR scanners [1] or structured light scanners [2]. However, structured light scanning is sometimes expensive and is viable under certain conditions. Another solution is to create 3D models directly from high-resolution camera images captured under favorable lighting conditions. One such solution is a multi-camera-based photogrammetric setup capturing a fixed-size volume. Such camera setups are typically calibrated and capture high-resolution static photos simultaneously. These camera setups produce high-quality 3D models and precise measurements. However, such a setup is also very expensive due to the requirement of special equipment such as multiple cameras, special light sources, and studio setups. A low-cost solution to this problem is Structure from Motion (SfM), which aims to create sparse 3D models using multiple images of the same object, captured from different viewpoints using a single camera, and without requiring camera locations and orientations.

SfM has become a popular choice to create 3D models due to its low-cost nature and simplicity. Structure from Motion is a very well-studied research problem. In early research works, Pollefeys et al. [3] developed a complete system to build a sparse 3D model of the scene from uncalibrated image sequences captured using a hand-held camera. At the time of writing, there is a plethora of choices for SfM software packages, each with its unique features and capabilities. Some are open-source software, such as COLMAP [4], MicMac [5], OpenMVS [6], and so on, while some others are commercial software packages, such as Metashape (https://www.agisoft.com (accessed on 23 May 2023)), RealityCapture (https://www.capturingreality.com (accessed on 23 May 2023)), etc. They rely on automatic keypoint detection and matching algorithms to estimate 3D structures. The input to such an SfM software is only a collection of digital photographs, generally captured by the same camera. However, these fully automatic tools usually require suitable lighting conditions and high-quality photographs, to generate high-quality 3D models. These conditions are very difficult to be fulfilled in industrial environments because there may be low lighting (which exacerbates blurring) and utility companies may have legacy video cameras capturing videos at low resolution. These legacy cameras are meant for plants’ visual inspection and enduring chemical, temperature, and radiation stresses.

**Figure 1:**
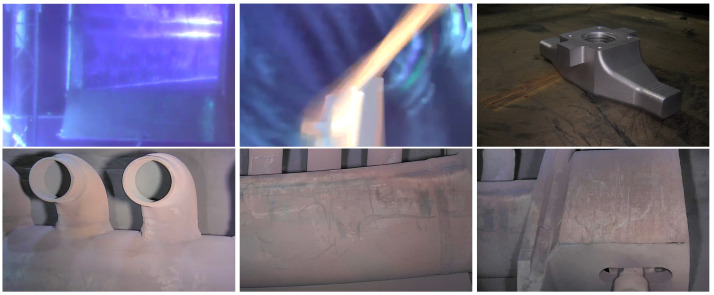
Examples of frames from videos captured in industrial environments. These videos are not suitable for automatic SfM tools due to issues such as low resolution, aggressive compression, strong and moving directional lighting (e.g., a torchlight mounted on the camera), motion blur, featureless surfaces, liquid turbulence, low lighting, etc.

The mentioned issues may become more severe in video-based SfM because video frames have motion blur and are aggressively compressed, leading to strong compression artifacts (e.g., ringing, blocking, etc.). Most modern cameras capture videos at 30 fps, so a few minutes of video produces a high number of frames, e.g., 10 min of footage is already 18,000 frames. Such a high number of frames not only increase computational time significantly but also give low-quality 3D output due to insufficient camera motion in consecutive frames. If we pass such featureless images (e.g., see Figure 1) as inputs to an SfM software, the number of accurately detected features and correspondences will be very low, leading to a low-quality 3D output. In this context, we have developed Movie Reconstruction Laboratory (MoReLab) (https://github.com/cnr-isti-vclab/MoReLab (accessed on 23 May 2023)), which is a software tool to perform user-assisted reconstruction on uncalibrated camera videos. MoReLab will address the problem of SfM in the case of featureless and poor-quality videos by exploiting the user indications about the structure to be reconstructed. A small amount of manual assistance can produce accurate models also in these difficult settings. User-assisted 3D reconstruction can significantly decrease the computational burden and also reduce the number of input images required for 3D reconstruction.

In contrast to automatic feature detection and matching-based SfM systems, the main contribution of MoReLab is a user-friendly interactive way that allows the user to provide topology prior to reconstruction. This modification allows MoReLab to achieve better results in featureless videos by leveraging the user’s knowledge of visibility and understanding of the video across frames. Once the user has added features and correspondences manually on 2D images, a bundle adjustment algorithm [7] is utilized to estimate camera poses and a sparse 3D point cloud corresponding to these features. MoReLab achieves accurate sparse 3D points estimation by adding features on as few as two or three images. The estimated 3D point cloud is overlaid on manually added 2D feature points to give a visual indication of the accuracy of estimated 3D points. Then, MoReLab provides several primitives such as rectangles, cylinders, curved cylinders, etc., to model parts of the scene. Based on a visual understanding of the shape of the desired object, the user selects the appropriate primitive and marks vertices or feature points to define it in a specific location. This approach gives control to the user to extract specific shapes and objects in the scene. By exploiting inputs from the user at several stages, it is possible to obtain 3D reconstruction even from poor-quality videos. Additionally, the overall computational burden with regard to a fully automatic pipeline is significantly reduced.

## 2. Related Work

There have been several research works in the field of user-assisted reconstruction from unordered and multi-view photographs. Early research works include VideoTrace [8], which is an interface to generate realistic 3D models from video. Initially, automatic feature detection-based SfM is applied to video frames, and a sparse 3D point cloud is overlaid on the video frame. Then, the user traces out the desired boundary lines, and a closed set of line segments generates an object face. Sinha et al. [9] modeled architectures using a combination of piecewise planar 3D models. Their system also computes sparse 3D data in such a way that lines are extracted, and vanishing points are estimated in the scene as well. After this automatic preprocessing, the user draws outlines on 2D photographs. Piecewise planar 3D models are estimated by combining user-provided 2D outlines and automatically computed sparse 3D points. A few such user interactions can create a realistic 3D model of the scene quickly. Hu et al. [10] developed an interface for creating accurate 3D models of complex mechanical objects and equipment. First, sparse 3D points are estimated from multi-view images and are overlaid on 2D images. Second, stroke-based sweep modeling creates 3D parts, which are also overlaid on the image. Third, the motion structure of the equipment is recovered. For this purpose, a video clip recording of the working mechanism of the equipment is provided, and a stochastic optimization algorithm recovers motion parameters. Rasmuson et al. [11] employ COLMAP [4] as a preprocessing stage to calibrate images. Their interface allows users to mark image points and place quads on top of images. The complete 3D model is obtained by applying global optimization on all quad patches. By exploiting user-provided information about topology and visibility, they are able to model complex objects as a combination of a large number of quads.

Some researchers developed interfaces where users can paint desired foreground regions using brush strokes. Such an interface was developed by Habbecke and Kobbelt [12]. Their interface consists of a 2D image viewer and a 3D object viewer. The user paints the 2D image in a 2D image viewer with the help of a stroke. The system computes an optimal mesh corresponding to the user-painted region of input images. During the modeling session, the system incrementally continues to build 3D surface patches and guide the surface reconstruction algorithm. Similarly, in the interface developed by Baldacci et al. [13], the user indicates foreground and background regions with different brush strokes. Their interface allows the user to provide localized hints about the curvature of a surface. These hints are utilized as constraints for the reconstruction of smooth surfaces from multiple views. Doron et al. [14] require stroke-based user annotations on calibrated images, to guide multi-view stereo algorithms. These annotations are added into a variational optimization framework in the form of smoothness, discontinuity, and depth ordering constraints. They show that their user-directed multi-view stereo algorithm improves the accuracy of the reconstructed depth map in challenging situations.

Another direction in which user interfaces need to be developed is single-view reconstruction. Single-view reconstruction is complicated without any prior knowledge or manual assistance because epipolar cannot be established. Töppe et al. [15] introduced convex shape optimization to minimize weighted surface area for a fixed user-specified volume in single-view 3D reconstruction. Their method relies on implicit surface representation to generate high-quality 3D models by utilizing a few user-provided strokes on the image. 3-Sweep [16] is an interactive and easy-to-use tool for extracting 3D models from a single photo. When a photo is loaded into the tool, it estimates the boundary contour. Once the boundary contour is defined, the user selects the model shape and creates an outline of the desired object using three painting brush strokes, one in each dimension of the image. By applying the foreground texture segmentation, the interface quickly creates an editable 3D mesh object which can be scaled, rotated, or translated.

Recently, researchers have made significant progress in the area of 3D reconstruction using deep learning approaches. The breakthrough work by Mildenahall et al. [17] introduced NeRF, which synthesizes novel views of a scene using a small set of input views. A NeRF is a fully connected deep neural network whose input is a single 5D coordinate (spatial location (x,y,z) and viewing direction (θ,ϕ)), and output is emitted radiance and volume density. To the best of our knowledge, a NeRF-like method that tackles at the same time all conditions of low-quality videos (blurred frames, low resolution, turbulence caused by liquids, etc.) have not been presented yet [18]. A GAN-based work, Pi-GAN [19], is a promising generative model-based architecture for 3D-aware image synthesis. However, their method has the main focus on faces and cars, so to be applicable in our context, there is the need to build a specific dataset for re-training (e.g., a dataset of industrial equipment, 3D man-made objects, and so on). Tu et al. [20] presented a self-supervised reconstruction model to estimate texture, shape, pose, and camera viewpoint using a single RGB input and a trainable 2D keypoint estimator. Although this method may be seminal for more general 3D reconstructions, the current work is currently focused on human hands.

Existing research works pose several challenges for low-quality industrial videos, which are typically captured by industrial utility companies. First, most works [8,9,10,11,14] in user-assisted reconstruction, still require high-quality images because they are using automatic SfM pipelines as their initial step. Our focus is on low-quality videos in industrial scenarios, where SfM generates an extremely sparse point cloud, making subsequent 3D operations extremely difficult. Second, these research works lack sufficient functionalities to be able to model a variety of industrial equipment. Third, these research works are not available as open-source, limiting their usage for non-technical users. Hence, our research contributions are as follows:

- A graphical user interface for the user to add feature points and correspondences manually to model featureless videos;
- Several primitive shapes to model the most common industrial components.

In MoReLab, there is no feature detection and matching stage. Instead, the user needs to add features manually based on the visual understanding of the scene. We have implemented several user-friendly functionalities to speed up this tedious process for the user. MoReLab is open-source software targeted for modeling industry scenarios and available for non-commercial applications for everyone.

## 3. Method

In this section, we describe the pipeline, the graphical user interface, and the primitive tools of MoReLab. We designed the software to be user-friendly and easy to use for new users. However, understanding the tools and design of this software will enable the user to achieve optimal results with MoReLab.

### 3.1. Graphical User Interface

**Figure 2:**
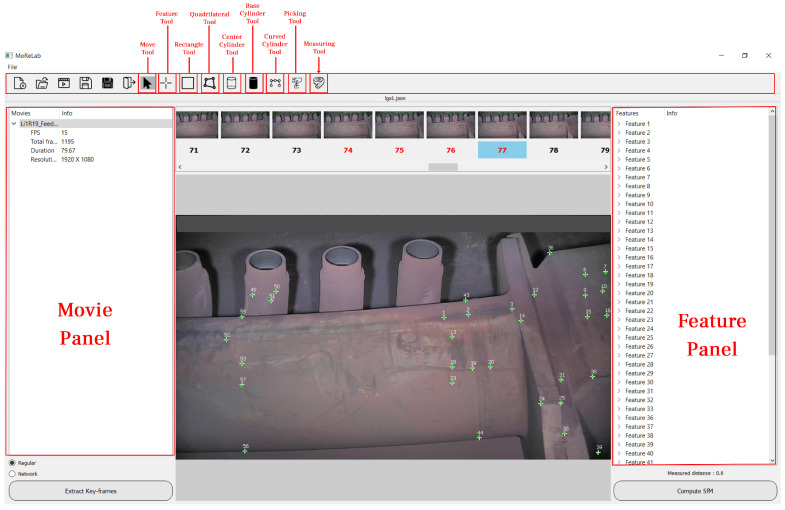
The graphical user interface of MoReLab. The toolbar at the top allows the user to switch between different tools.

Figure 2 shows the graphical user interface (GUI) of MoReLab. The user starts the 3D modeling process by importing a video, which is loaded into the movie panel. Then, by clicking on the ‘Extract Key-frames’ button, the extracted keyframes would appear in the central top scroll bar area. The user can click on the thumbnail, and display the corresponding image in the central area. At this point, it is possible to use the ‘Feature Tool’ to add features to the image with a mouse double-click at the desired location. A white-colored plus-shaped feature appears on the image, and the information about the feature will appear in the right feature panel. Information includes the associated frame and the feature location. Once the user has marked features, the ‘Compute SfM’ can be launched. This option will perform bundle adjustment and calculate the 3D structure. 3D points are visualized on the image as green-colored points. Figure 2 shows estimated 3D points that are approximately at the same locations as marked 2D features. Once 3D points have been estimated, the user can make use of the shape tools, i.e., the rectangle tool, quadrilateral tool, center cylinder tool, base cylinder tool, and curved cylinder tool, to model different shapes. The picking tool allows the user to select and delete different primitives. Finally, the measuring tool allows the user to calibrate 3D data points and perform measurements.

### 3.2. Pipeline

**Figure 3:**
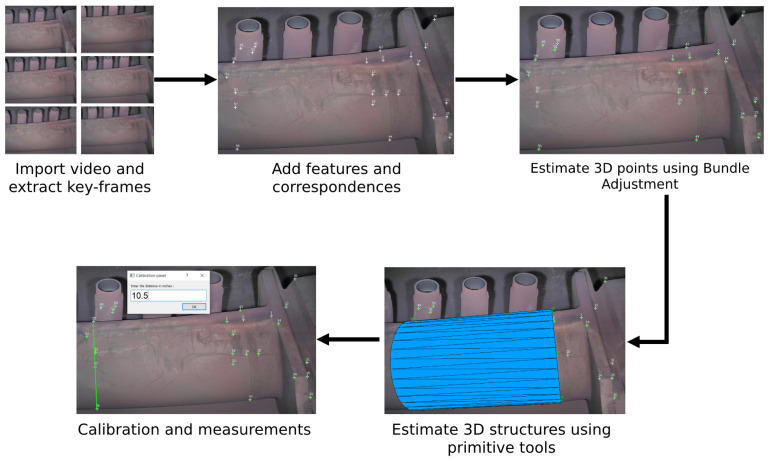
MoReLab reconstruction pipeline.

Figure 3 presents the pipeline of our software. This pipeline consists of the following steps:

#### 3.2.1. Manual Feature Extraction

In the second step, the user grabs the feature tool and starts to add features. A feature refers to an identifiable and distinctive pattern, shape, color, texture, or point of interest in an image. The user needs to choose only a few frames based on the recognizability of features. Since we are using the eight-point algorithm [21] to compute the fundamental matrix in the next step, the user needs to add a minimum of eight features in at least two frames. However, increasing the number of features and adding features on more views would increase computational accuracy. To speed up this tedious process, the user can copy the location of all features on an image with a simple keyboard press and paste features at pixel coordinates on other keyframes. Each feature location can be adjusted by dragging it to the correct location.

#### 3.2.2. Extract Keyframes

In the first step, a video is loaded into the software, and frames are extracted. However, all frames are not required because of several reasons. First, processing all frames is computationally very expensive. Second, some video frames have motion blur, making it difficult for the user to add features. Third, a very small baseline between consecutive frames causes inaccurate triangulation and reconstruction. We implemented two methods of keyframe extraction in MoReLab: The first approach is to regularly sample frames at a desired frequency, and the second approach is based on a network [22].

This latter method automatically removes out-of-focus frames, blurred frames, and redundant frames (i.e., due to a static scene). In addition, it selects frames that may lead to a high-quality reconstruction. Note that other frame selection methods can be employed such as Nocerino et al. [23].

We designed a simple calibration panel containing a combo box to switch easily between both approaches. The first approach is faster than the latter.

#### 3.2.3. Bundle Adjustment

In the third step, feature locations provided by the user are utilized to compute a sparse 3D point cloud through bundle adjustment. bundle adjustment is the process of refining camera parameters and 3D point locations simultaneously, by minimizing the re-projection error between input 2D locations and projected 2D locations of 3D points on the image. The minimization algorithm being used is the Trust Region Reflective Algorithm [24]. Assume that *n* 3D points can be observed in *m* views. Let $x_{ij}$ denote the *i*-th feature location on hte *j*-th image, $X_{i}$ denote the corresponding *i*-th 3D point, and $C_{j}$ denote the camera parameters corresponding to the *j*-th image, then the objective function for bundle adjustment can be defined as:

(1) $$\arg\min_{X_{i},C_{j}}\sum_{i=1}^{n}\sum_{j=1}^{m}b_{ij}d\left(f\left(X_{i},C_{j}\right),x_{ij}\right),$$

where $b_{ij}$ denotes a binary variable that equals 1 if the feature *i* is visible on the image *j* and 0 otherwise. $f(X_{i},C_{j})$ is the projection of *i*-th 3D point on *j*-th image. $d\left(f\left(X_{i},C_{j}\right),x_{ij}\right)$ indicates the Euclidean distance between the projection point and $x_{ij}$. After this minimization, we obtain optimal camera parameters and locations of 3D points in the world coordinate frame.

#### 3.2.4. Primitive Tools

We have implemented tools based on geometric primitive shapes, to be able to model a variety of industrial equipment. These tools are described as follows:

- **Rectangle Tool:** This tool allows the user to model planar surfaces. To estimate a rectangle, the user should click on four features in an anti-clockwise manner. The 3D sparse points, corresponding to four selected features, compute new vertices to form a rectangle in which all inner angles are constrained to be 90 degrees.
- **Quadrilateral Tool:** This tool allows creating a quadrilateral using four 2D features. This tool connects 3D sparse points corresponding to selected 2D features. Unlike the rectangle tool, there is no 90-degree angle constraint and opposite sides might not be parallel. Hence, inner angles might not be 90 degrees. If the selected four points are not in a single plane, a quadrilateral is also not planar.
- **Center Cylinder Tool:** This tool models a cylindrical object using a specific point. This is useful when the center of the base of cylindrical equipment is visible. The user needs to click on four points. The point can be either a 2D feature or an area containing a 3D primitive. For 2D features, we get the corresponding 3D sparse point computed from bundle adjustment. The initial three points form the base of the cylinder, and the fourth point determines the height of the cylinder. The first point corresponds to the center of the base, the second point forms an axis point, and the third point corresponds to the radius of the cylinder.
  A cylinder is estimated by computing new axes. Let us denote input 3D point data points as P1, P2, P3, and P4. We define a reference system:
  (2) $$T=\frac{P2-P1}{∥P2-P1∥}\;b=P3-P1\;N=\frac{T\times b}{∥T\times b∥}\;B=T\times N,$$
  where N is the normal, B is the bi-normal, and T is the tangent. ∥b∥ is the radius of the cylinder, and the base of the cylinder lies in the plane formed by T and B axes. The height of the cylinder is calculated by projecting the vector P4−P1 on N.
- **Base Cylinder Tool:** This tool allows users to create a cylinder in which the initial three selected points lie on the base of the cylinder. The fourth point determines the height of the cylinder. This is useful for most industrial scenarios because, in most cases, we can only see the surface of the cylindrical equipment, and the base center is not visible. As in other tools, the user needs to select the points by clicking on them. The point can be either a 2D feature or an area containing a 3D primitive. For 2D features, we get the corresponding 3D sparse point computed from bundle adjustment.
  Similar to the center cylinder tool, first, we need to calculate a new local axes system, i.e., T, B, and N similar to how these axes were calculated in the center cylinder tool. In the new local system, the first point is considered to be at the origin; while the second and third 3D points are projected on B and T to obtain their 2D locations in the plane formed by B and T. Given these three 2D points, we find the circle passing through these three points. If three points are in a straight line, the circle would not be estimated because it would have an infinite radius. Once we know the center and radius of this circle, we calculate the base and top points, similar to the center cylinder tool.
- **Curved Cylinder Tool:** This tool models curved pipes and curved cylindrical equipment. The user clicks on four points at any part of the image. Then, the user clicks on a sparse 3D point obtained from bundle adjustment, this last point assigns an approximate depth to the curve just defined. To do this, first, we estimate the plane containing this 3D point, denoted as P. Typically, a plane is defined as:
  (3) ax+by+cz+d=0,
  where coefficients *a*, *b*, and *c* can be obtained from the *z*-vector of a camera projection matrix, *M*. *d* is obtained by the dot product of the *z*-vector and P. Assume that s represents the 2D point clicked by the user at (x,y) coordinates on the image and X represents the unknown 3D point corresponding to s.
  (4) $$M=\left[\begin{array}{c} M_{1}\\ M_{2}\\ M_{3}\\ M_{4} \end{array}\right]\;x=\frac{M_{1}X}{M_{3}X}\;y=\frac{M_{2}X}{M_{3}X}\;\left[a\;b\;c\right]\cdot X+d=0.$$
  Equation (4) can be re-arranged into the form of linear equation AX = b and a linear solver finds X. Through this procedure, four 3D points are obtained corresponding to the clicked points on the frame. These four 3D points act as control points to estimate a Bézier curve [25] on the frame. Similarly, the user can define the same curve from a different viewpoint. These curves defined at different viewpoints are optimized to obtain the final curve in 3D space. This optimization is about minimizing the sum of the Euclidean distance between control points across frames and the Euclidean distance between the location of the projected point and the location of the 2D feature in each frame containing the curve.
  Assume that *m* frames contain curves. Let $x_{ij}$ denote the *i*-th feature location on the *j*-th image, ${\mathrm{CP}}_{ij}$ denotes *i*-th control point on the *j*-th frame. $X_{i}$ denotes corresponding *i*-th 3D point, and $C_{j}$ denotes camera parameters corresponding to *j*-th image, then the objective function for optimization of curves is defined as:
  (5) $$\arg\min_{{\mathrm{CP}}_{ij}}\;\sum_{j=1}^{m-1}CP_{j}-CP_{j+1}+\sum_{j=1}^{m}\sum_{i=1}^{4}d\left(f\left({\mathrm{CP}}_{ij},C_{j}\right),x_{ij}\right),$$
  where $f({\mathrm{CP}}_{ij},C_{j})$ is the projection of the *i*-th control point on the *j*-th image. The Euclidean distance between the projected point and $x_{ij}$, is represented by $d\left(f\left({\mathrm{CP}}_{ij},C_{j}\right),x_{ij}\right)$.
  The optimal control points, obtained from optimization, estimate the final Bézier curve and the cylinder needs to be built around this curve. In order to define the radius of this curved cylinder, the user clicks on a 3D point, and a series of cylinders are computed around the final curve.

#### 3.2.5. Calibration and Measurements

Taking real-world measures on the reconstructed object is important in industrial scenarios. For example, the 3D reconstruction can be used to evaluate if a pipe or other objects have been deformed and then make the necessary maintenance/actions. The measurement tools allow the user to measure the distance between two 3D points. These points can be in any primitive, i.e., quad, cylinder, or simple 3D point.

The sparse point cloud obtained from bundle adjustment cannot be used directly to get real-world measurements because the camera is calibrated up to a scale factor. Hence, first, the user needs to assign the proper scale between two 3D points. In this step, the user draws a line between two 3D points, and a simple panel opens up and asks the user to input the corresponding known distance. This ground-truth distance is employed to calculate a distance scaling factor. The second step is the actual measurement, in which the user can draw a line between any 3D points, and MoReLab calculates the corresponding properly scaled distance using the scaling factor.

## 4. Experiments and Results

We analyzed the performance of MoReLab and other approaches on some videos for modeling different industrial equipment. We started our comparison using an image-based reconstruction software package, showing that the results are of poor quality in these cases. Then, we will show what we obtain with user-assisted tools for the same videos. We performed our experiments on two datasets. The first dataset consists of videos provided by a utility company in the energy sector. Ground-truth measurements have also been provided for two videos of this dataset for quantitative testing purposes. The second dataset was captured in our research institute to provide some additional results.

Agisoft Metashape is a popular high-quality commercial SfM software, which we applied to our datasets. Such software extracts features automatically, matches them, calibrates cameras, densely reconstructs the final scene, and generates a final mesh. The output mesh can be visualized in a 3D mesh processing software such as MeshLab [26].

Results obtained with SfM software allow us to model these videos with user-assisted tools, e.g., see Figure 7b. 3-Sweep is an example of software for user-assisted 3D reconstruction from a single image. It requires the user to have an understanding of the shapes of the components. Initially, the border detection stage uses edge detectors to estimate the outline of different components. The user selects a particular primitive shape, and three strokes generate a 3D component that snaps to the object outline. Such a user-interactive interface combines the cognitive abilities of humans with fat image processing algorithms. We will perform a visual comparison of modeling different objects with an SfM software package, 3-Sweep, and our software. Table 1 presents a qualitative comparison of the functionalities of software packages being used in our experiments. The measuring tool in MeshLab performs measurements on models exported from Metashape and 3-Sweep.

### 4.1. Cuboid Modeling

**Figure 4:**
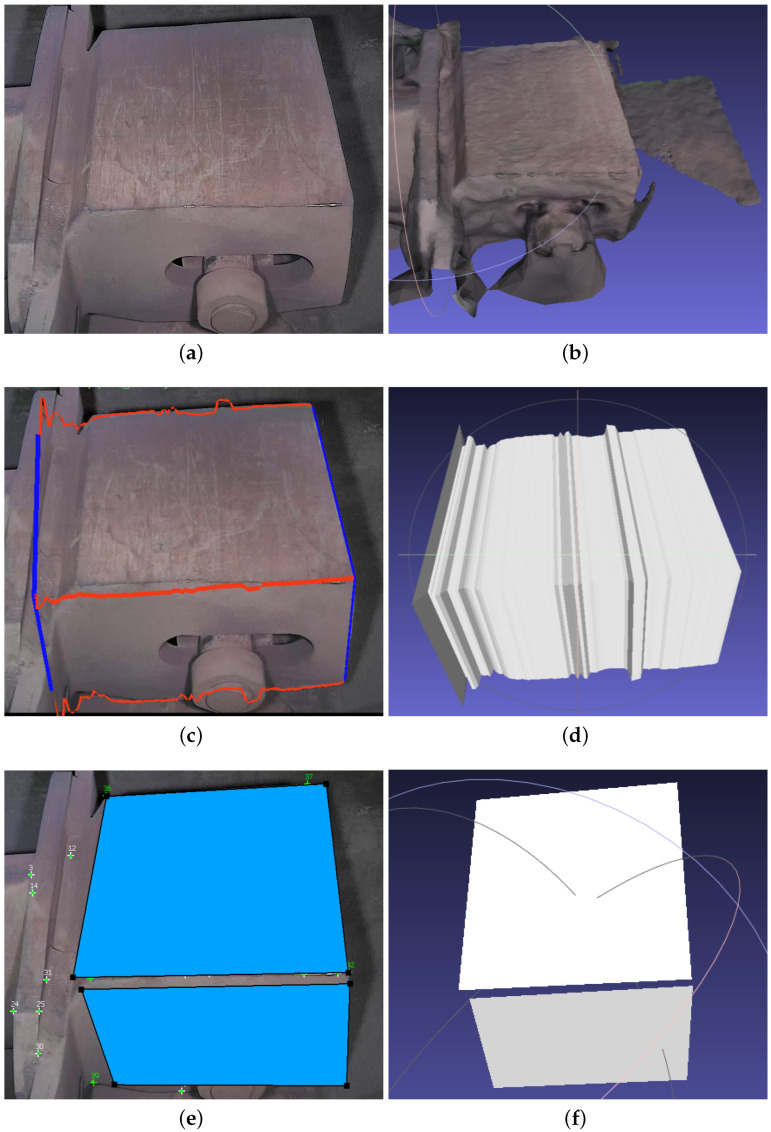
Modeling a cuboid with Metashape, 3-Sweep, and MoReLab: (**a**) A frame of input video; (**b**) Cuboid modeling with Metashape; (**c**) Paint strokes snapped to cuboid outline; (**d**) Cuboid modeling with 3-Sweep; (**e**) Modeling with rectangle tool; (**f**) MeshLab visualization of estimated surfaces of the cuboid.

3-Sweep allows us to model cuboids. In MoReLab, flat 2D surfaces can be modeled with the rectangle tool and quadrilateral tool. To estimate a cuboid, more rectangles and quadrilaterals need to be estimated in other views as well to form a cuboid. Figure 4 shows the results of modeling an image segment containing a cuboid with Metashape, 3-Sweep, and MoReLab. Figure 4b shows the result of the approximation of the cuboid with Metashape. There is a very high degree of approximation and the surface is not smooth.

Figure 4c,d show the result of extracting a cuboid using 3-Sweep. The modeling in 3-Sweep starts by detecting the boundaries of objects at the start. Despite changing thresholds, this detection stage is prone to errors and shows very little robustness. Hence, the boundary of the extracted model is not smooth, and the shape of the model is irregular. Figure 4e,f illustrate modeling in MoReLab using the rectangle tool and quadrilateral tool. Every rectangle in Figure 4e is planar with 90-degree inner angles. However, each quadrilateral in Figure 4f may or may not be planar, depending on the 3D locations of the points being connected. In other words, orthogonality is not enforced in Figure 4f.

### 4.2. Jet Pump Beam Modeling

**Figure 5:**
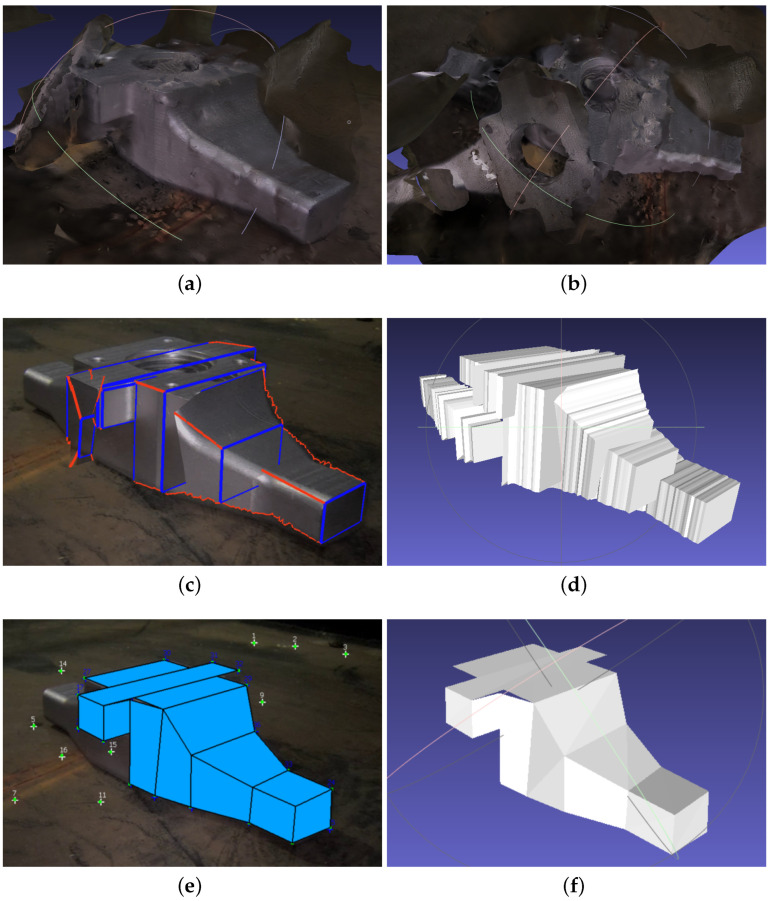
Jet pump beam is modeled with tested software programs under consideration: (**a**) Metashape reconstruction output; (**b**) Another view of (**a**); (**c**) Paint strokes snapped to jet pump beam outline; (**d**) Output obtained by modeling jet pump beam with 3-Sweep; (**e**) Estimation of jet pump beam surface using quadrilateral tool in MoReLab; (**f**) Output obtained by modeling jet pump beam with MoReLab.

The jet pump beam is monitored in underwater and industrial scenarios, to observe deformations or any other issues. The jet pump beam is also modeled with different software programs in Figure 5. Metashape reconstructs a low-quality 3D model of the jet pump beam. Another view of Figure 8a shows that Metashape has estimated two jet pump beams instead of a single jet pump beam. The beam model is passing through the floor in this reconstruction. The jet pump beam model is missing surfaces at different viewpoints, and the model is merged with the floor at different places. This low-quality result can be attributed to dark environments, the featureless surface of the pump, and the low distance of the object from the camera. The mesh, obtained by modeling the jet pump beam with 3-Sweep, has a low-quality boundary and does not represent the original shape of the jet pump beam (see Figure 5d).

The jet pump beam has also been modeled with MoReLab in Figure 5e. The quadrilateral tool has been used to estimate the surface of the jet pump beam. The output mesh is formed by joining piecewise quadrilaterals on the surface of the jet pump beam. Quadrilaterals on the upper part of the jet pump are aligned very well together; but, some misalignment can be observed on surfaces at the side of the jet pump beam. The resulting mesh has a smooth surface and reflects the original shape of the jet pump beam. Hence, this result is better than the mesh in Figure 5b and mesh in Figure 5d.

### 4.3. Cylinder Modeling

**Figure 6:**
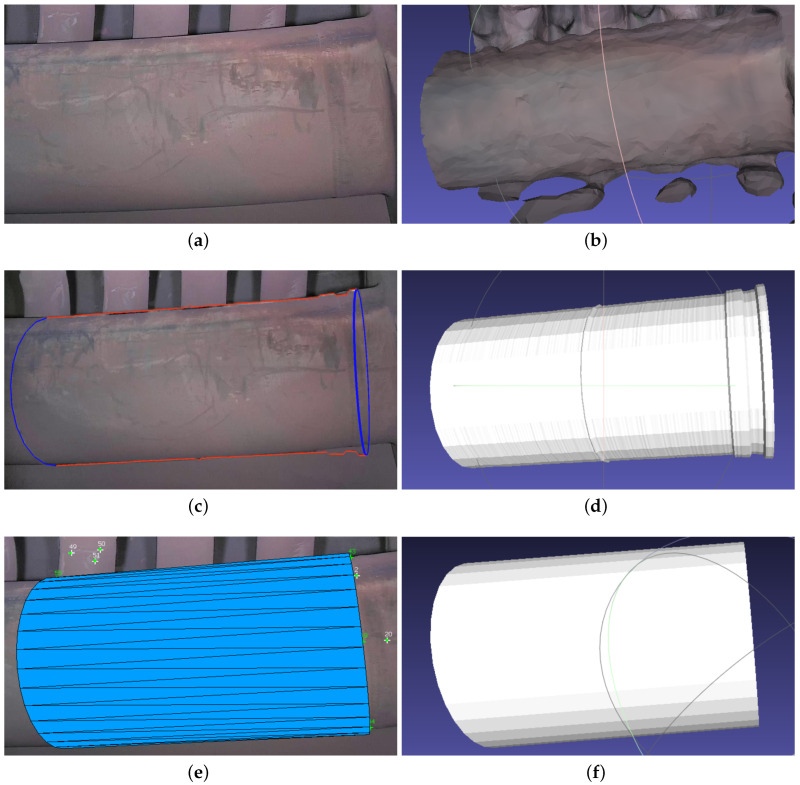
An example of modeling a cylinder with Metashape, 3-Sweep, and MoReLab: (**a**) A frame of input video; (**b**) MeshLab visualization of a cylinder created using Metashape; (**c**) Paint strokes snapped to cylinder outline in 3-Sweep; (**d**) MeshLab visualization of a cylinder modeled using 3-Sweep; (**e**) Modeling a cylinder using base cylinder tool in MoReLab; (**f**) MeshLab visualization of a cylinder mesh obtained from MoReLab.

Equipment of cylindrical shape is common in different industrial plants. We have also modeled a cylinder with our tested approaches, and the results have been presented in Figure 6. In the Metashape reconstruction of the cylinder in Figure 6b, some geometric artifacts are observed, and the surface is not smooth. Figure 6c,d show the result of using 3-Sweep. While the boundary detection is better than that in Figure 4c, the cylinder still does not have a smooth surface. On the other hand, the cylinder mesh obtained by modeling with MoReLab has a smoother surface and is more consistent than that obtained with 3-Sweep.

Figure 6e,f show the result of modeling a cylinder, using the base cylinder tool in MoReLab. The reason to use this specific tool is that the center of the cylinder base is not visible, and features are visible only on the surface of the cylinder. As just stated, the cylinder obtained is more consistent and smooth than the one obtained with Metashape and 3-Sweep.

### 4.4. Curved Pipe Modeling

**Figure 7:**
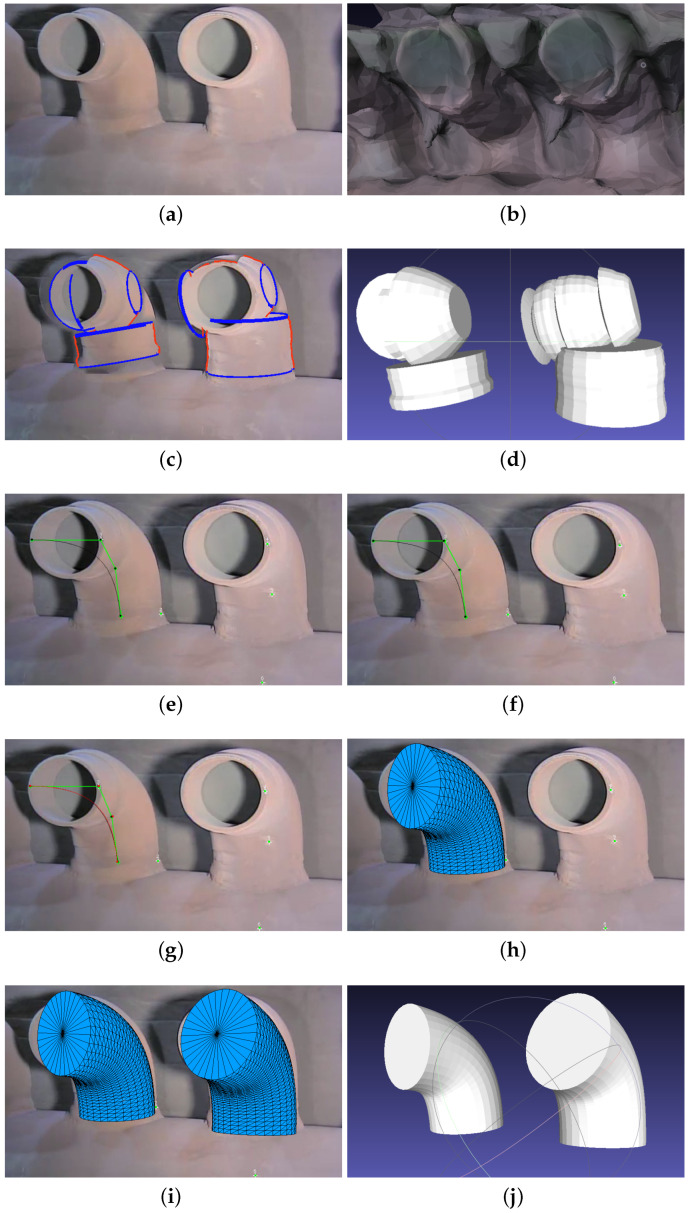
An example of modeling a curved pipe: (**a**) A frame of input video; (**b**) Modeling curved pipes in Metashape; (**c**) Paint strokes snapped to curved cylinder outlines; (**d**) Estimation of curved pipes using 3-Sweep visualized in MeshLab; (**e**) Bézier curve is drawn on a frame; (**f**) Bézier curve is drawn on another frame; (**g**) Curves on multiple frames are optimized to obtain the final Bézier curve shown by red color; (**h**) A cylinder around the curve is created; (**i**) A copy of the first cylinder is placed on the second pipe; (**j**) Estimated curved cylinders are visualized in MeshLab.

Figure 7 compares the modeling of curved pipes in Metashape, 3-Sweep, and MoReLab. In general, the reconstruction of curved pipes is difficult due to the lack of features. Figure 7b shows the result of modeling curved pipes using Metashape. The result is extremely low-quality because background walls are merged with the pipes, and visually similar pipes produce different results. The result of using 3-Sweep is shown in Figure 7c. As shown in Figure 7d, the mesh obtained with 3-Sweep hardly reflects the original pipe. Due to discontinuous outline detection and curved shape, multiple straight cylinders are estimated to model a single curved pipe.

Figure 7e–j show the steps of modeling two curved cylinders in MoReLab. The results are quite good, even if there is a small misalignment between the pipes and the underlying frame.

### 4.5. Additional Experiments

After observing the results of the data provided by the utility company, we captured a few more videos to conduct additional experiments and better evaluate our approach. These videos are captured on the roof of our research institute, which is full of steel pipes and other featureless objects.

**Figure 8:**
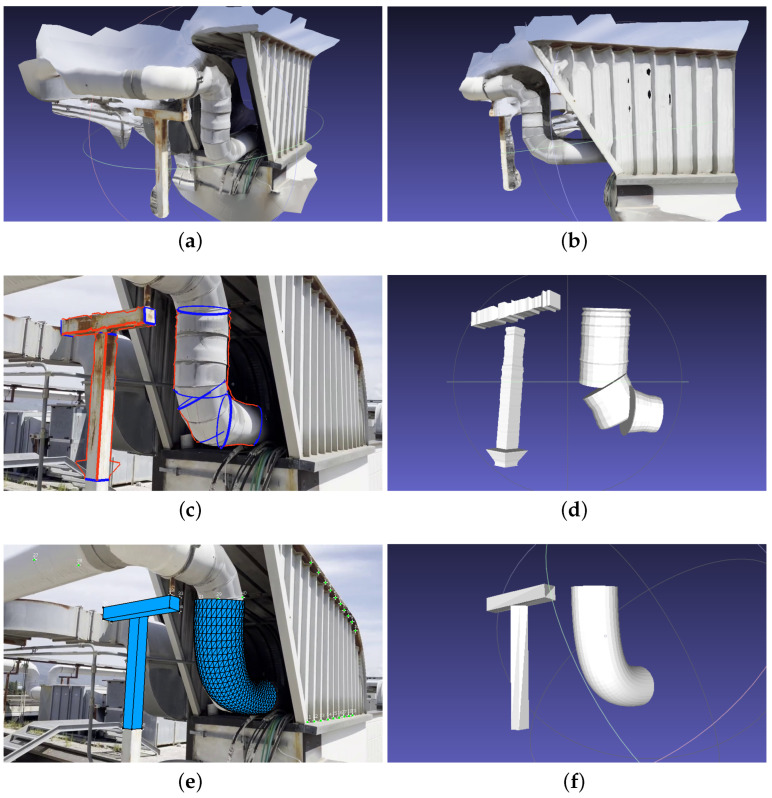
Modeling cuboids and a curved pipe with tested software programs: (**a**) Metashape reconstruction output visualized in MeshLab; (**b**) A different view of the Metashape reconstruction visualized in MeshLab; (**c**) Paint strokes snapped to desired object outlines; (**d**) 3-Sweep output visualized in MeshLab; (**e**) Estimation of desired objects in MoReLab; (**f**) Estimated objects are visualized in MeshLab.

Figure 8 shows the result of modeling a video with Metashape, 3-Sweep, and MoReLab. While the overall 3D model obtained with Metashape (Figure 8a) looks good, a visual examination of the same model from a different viewpoint (Figure 8b) shows that the T-shaped object and curved pipe lack a surface from behind. This can be due to the lack of a sufficient number of features and views at the back side of the T-shaped object and curved pipe. 3-Sweep output in Figure 8d shows gaps in 3D models of T-shaped object and curved pipe. As shown in Figure 8e,f, MoReLab is able to model desired objects more accurately, and a fine mesh can be exported easily from MoReLab.

**Figure 9:**
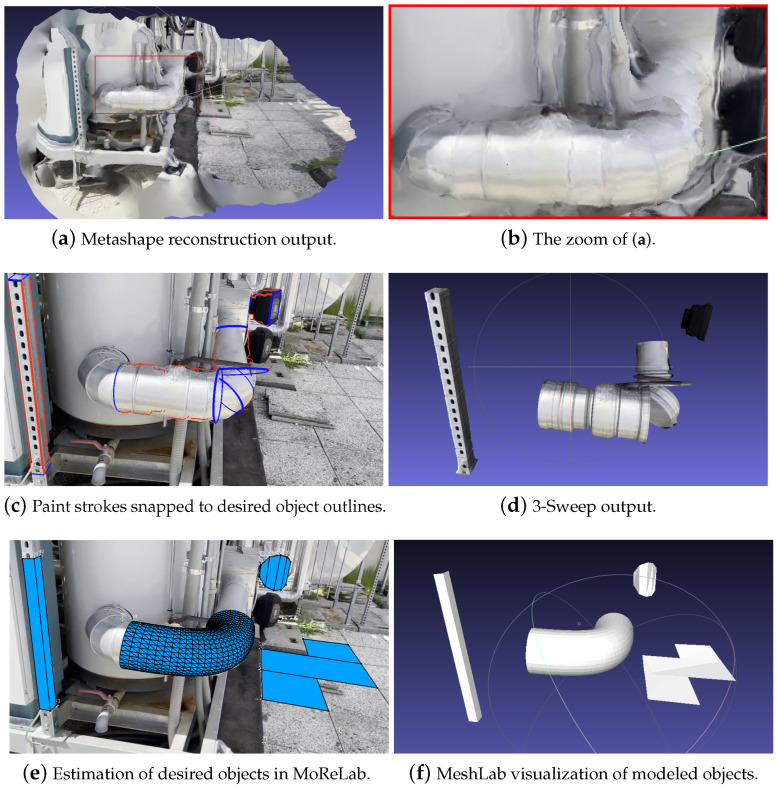
Modeling cuboids and a curved pipe with Metashape, 3-Sweep, and MoReLab.

Figure 9 shows the result of modeling another video. Metashape output (see Figure 9a) shows a high level of approximation. The red rectangular region represents the curved pipe in the frame, and Figure 9b shows the zoom-in of this rectangular region. The lack of a smooth surface reduces the recognizability of the pipe and introduces inaccuracies in the measurements. Figure 9d shows gaps in the 3D output model of a curved pipe. However, outputs obtained with MoReLab are more accurate and represent the underlying objects more accurately.

### 4.6. Discussion

The results obtained with SfM packages (e.g., see Figure 4b, Figure 6b, Figure 7b, Figure 8b, and Figure 9a) elicit the need to identify features manually and develop software for user-assisted reconstruction. The reason for low-quality output models obtained using 3-Sweep can be attributed to low-quality border detection. This is due to dark light conditions in these low-resolution images. 3-Sweep modeled high-resolution images in their paper and reported high-quality results in their work for high-quality images. However, our experiments indicate that 3-Sweep is not suitable for low-resolution images and industrial scenarios mentioned in Figure 1. In these difficult scenarios, 3-Sweep suffers from low robustness and irregularity in the shapes of meshes.

MoReLab does not rely on the boundary detection stage and hence generates more robust results. After computing sparse 3D points on the user-provided features, our software provides tools to the user to quickly model objects of different shapes. Figure 4f, Figure 5e, Figure 6e, Figure 7i, Figure 8e, and Figure 9e demonstrate the effectiveness of our software by showing the results obtained with our software tools.

### 4.7. Measurement Results

Given the availability of ground-truth data for two videos in the first dataset, we performed a quantitative analysis. The evaluation metric being used for quantitative analysis, is a relative error, $E_{\mathrm{rel}}$:

(6) $$E_{\mathrm{rel}}=100\times \left(\frac{|M_{e}-M_{g}|}{M_{g}}\right),$$

where $M_{g}$ is the ground-truth measurement, and $M_{e}$ is a measure length from the estimated 3D model.

#### 4.7.1. 1-Measurement Calibration

**Figure 10:**
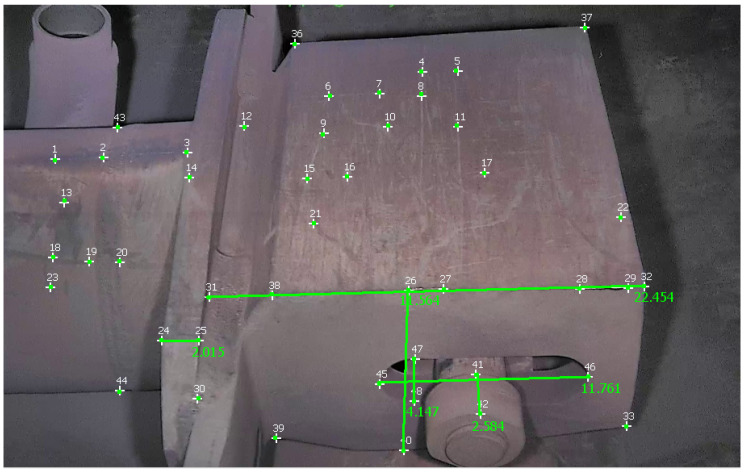
Measurements are taken in MoReLab. The distance of 22.454 cm between features 31 and 32 is the measurement provided for calibration. The other distances are calculated according to this reference distance.

In this section, we perform calibration with one ground-truth measurement. In all experiments, the longest measurement was taken as ground truth to have a more stable reference measure. This helps in mitigating the error of the calculated measurements. Table 2 reports measurements obtained with the different approaches on a video of the first dataset, and Figure 10 shows these measurements taken in MoReLab.

The selection of measurements has been done according to the available ground-truth measurements from diagrams of equipment. Table 2 also presents a comparison of relative errors with these three software packages. Among the five measurements under consideration, MoReLab achieves the lowest errors in three measurements and the lowest average relative error.

**Figure 11:**
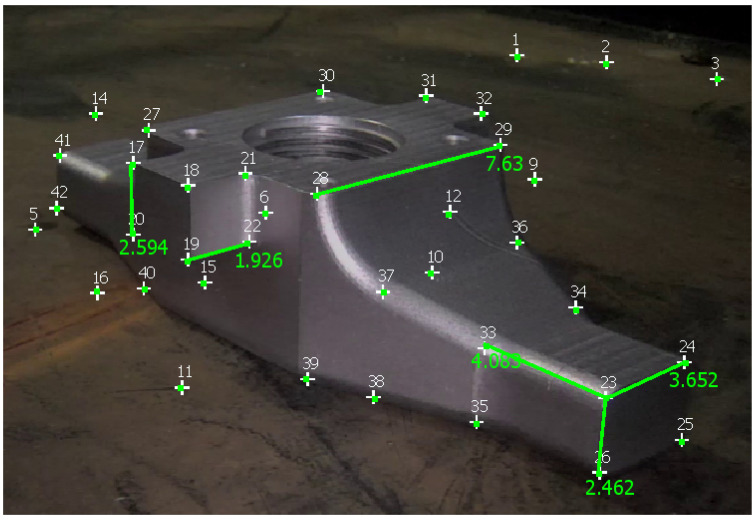
Measurements computed in MoReLab. The distance of 7.630 cm between features 28 and 29 is the measurement provided for calibration, and other distances are calculated.

Table 3 reports measurements obtained with Metashape, 3-Sweep, and MoReLab on another video of the first dataset, and Figure 11 shows these measurements taken in MoReLab. Given the availability of a CAD model for the jet pump, we take meaningful measurements between corners in a CAD file and use these measurements as ground truths. Table 3 also presents a comparison of relative errors with these three software packages. Among the five measurements under consideration, MoReLab achieves the lowest errors in three measurements and the lowest average relative error.

**Figure 12:**
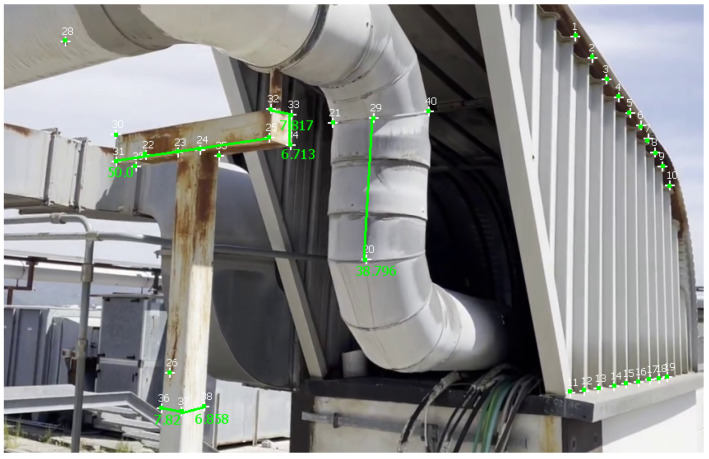
Measurements computed in MoReLab. The distance of 50 cm between features 25 and 31 is the measurement provided for calibration, and other distances are calculated.

Table 4 reports measurements and calculations for a video of the second dataset, and Figure 12 illustrates these measurements in MoReLab. We took some meaningful measurements to be used as ground truth for measurements with Metashape, 3-Sweep, and MoReLab. Relative errors are also calculated for these measurements and reported in Table 4. All software programs have achieved more accurate measurements in this video with respect to videos of the first dataset. This can be due to more favorable lighting conditions and high-resolution frames containing a higher number of recognizable features. Similar to Table 2 and Table 3, five measurements have been considered and MoReLab achieves the lowest relative errors in three measurements and the lowest average relative error in comparison to other software programs.

**Figure 13:**
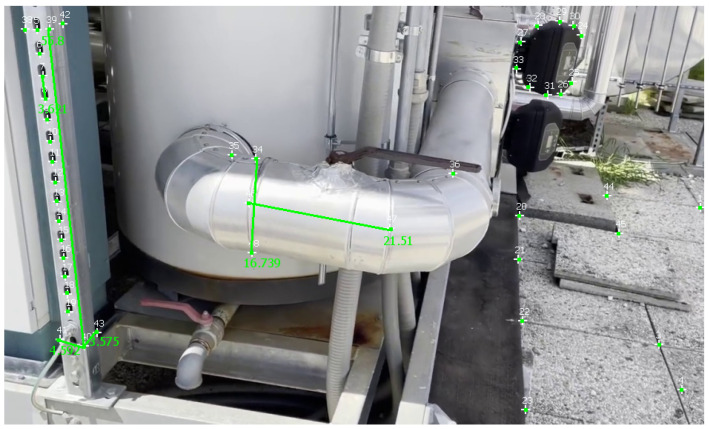
Measurements computed in MoReLab. The distances of 55.8 cm between features 39 and 40 are provided for calibration, and other distances are calculated.

Table 5 reports measurements obtained with Metashape, 3-Sweep, and MoReLab on another video of the second dataset, and Figure 13 illustrates these measurements in MoReLab. Among the five measurements under consideration, MoReLab achieves minimum error in four measurements and the lowest average relative error.

#### 4.7.2. Three-Measurement Calibration

To assess the robustness of the results presented now, we re-ran them by using the calibration factor for the measurements of the average of three calibration factors computed on three different measures. After the three-measurement calibration, we re-measured the distances in our four videos. Table 6, Table 7, Table 8 and Table 9 report measurements and their relative errors, where the three largest distances have been provided as calibration values for each video. Such results confirm the trend that we had before in Table 2, Table 3, Table 4 and Table 5, which have a single measurement for calibration. This trend shows that MoReLab provides less relative error on average than using 3-Sweep and Metashape for the 3D reconstruction of industrial equipment and plants.

#### 4.7.3. Limitations

From our evaluation, we have shown that our method performs better than other approaches for our scenario of industrial plants. However, users need to be accurate and precise when adding feature points and to use a high-quality methodology when performing measurements. Overall, all image-based 3D reconstruction methods, including ours, cannot achieve a precision of millimeters (at our scale) or less for many factors (e.g., sensor resolution). Therefore, if an object has a small scale the error introduced by the tolerance is lower than the reconstruction error.

## 5. Conclusions

We have developed a user-interactive 3D reconstruction tool for modeling low-quality videos. MoReLab can handle long videos and is well-suited to model featureless objects in videos. It allows the user to load a video, extract frames, mark features, estimate the 3D structure of the video, add primitives (e.g., quads, cylinders, etc.), calibrate, and perform measurements. These functionalities lay the foundations of the software and present a general picture of its use. MoReLab allows users to estimate shapes that are typical of industrial equipment (e.g., cylinders, curved cylinders, etc.) and measure them. We evaluated our tool for several scenes and compared results against the automatic SfM software program, Metashape, and another modeling software, 3-Sweep [16]. Such comparisons show that MoReLab can generate 3D reconstructions from low-quality videos with less relative error than these state-of-the-art approaches. This is fundamental in the industrial context when there is the need to obtain measurements of objects in difficult scenarios, e.g., in areas with chemical and radiation hazards.

In future work, we plan to extend MoReLab tools for modeling more complex industrial equipment and to show that we are not only more effective than other state-of-the-art approaches in terms of measurement errors but also more efficient in terms of the time that the user needs to spend to achieve an actual reconstruction. 

## Figures and Tables

**Figure 14:**
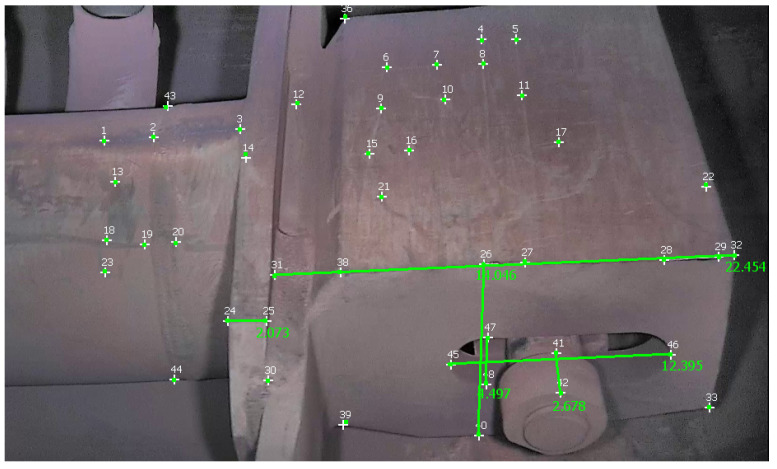
Measurements computed in MoReLab. The distances of 22.454, 14.046, and 12.395 cm are provided for calibration, and other distances are calculated.

**Figure 15:**
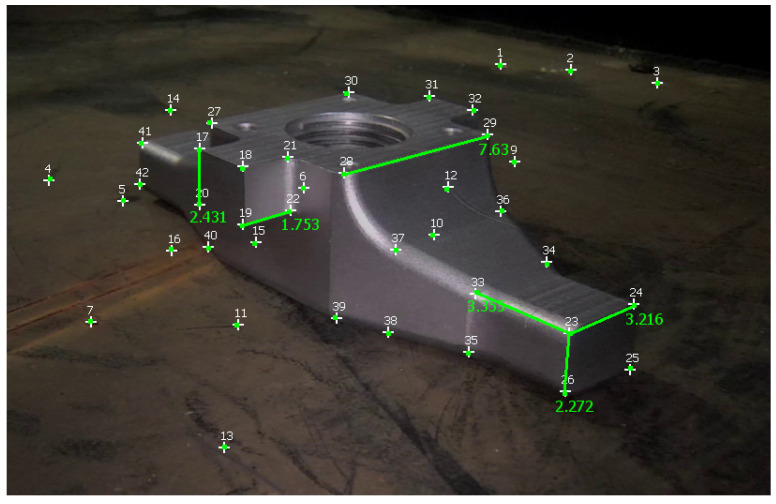
Measurements computed in MoReLab. The distances of 7.63, 3.355, and 3.216 cm are provided for calibration, and other distances are calculated.

**Figure 16:**
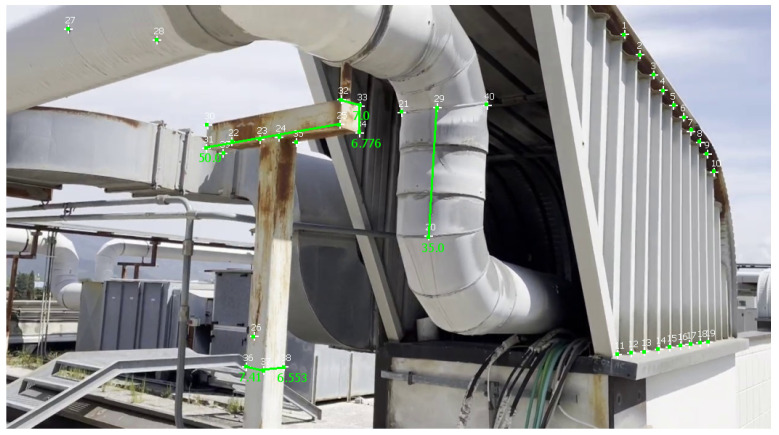
Measurements computed in MoReLab. The distances of 50, 35, and 7 cm are provided for calibration, and other distances are calculated.

**Figure 17:**
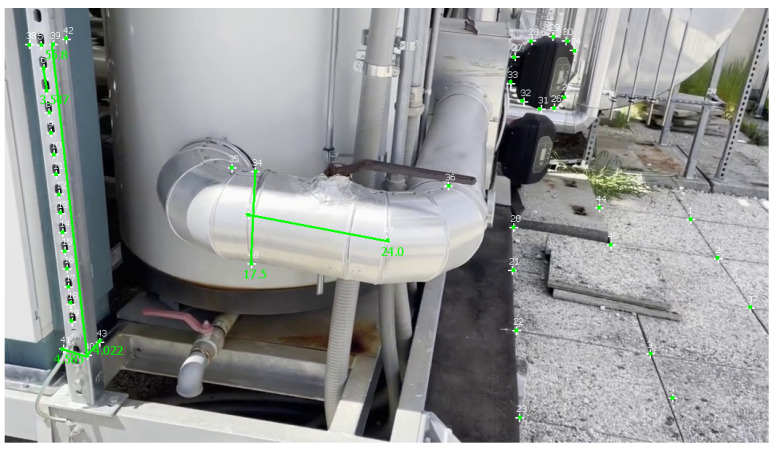
Measurements computed in MoReLab. The distances of 55.8, 24, and 17.5 cm are provided for calibration, and other distances are calculated.

**Table 1:** Qualitative comparison of the functionalities of different software packages.

	Automatic Feature Matching	Bundle Adjustment	Rectangle/Cylinder	Curved Cylinder	Measurements
Metashape	✓	✓	✗	✗	✓
3-Sweep	✗	✗	✓	✗	✗
MoReLab	✗	✓	✓	✓	✓

**Table 2:** Results of comparing MoReLab against Metashape and 3-Sweep in terms of relative error in measurements on the first video (see Figure 10).

Method	Ground Truth (cm)	Measured Distance (cm)	Relative Error
Metashape	14.046	13.472	4.087
	12.395	9.809	20.857
	4.115	2.664	35.201
	2.616	6.644	23.136
	2.057	1.852	9.889
		Average Relative Error	18.634
3-Sweep	14.046	13.858	**1.447**
	12.3953	12.669	**2.213**
	4.115	4.475	8.765
	2.616	3.731	42.621
	2.057	1.338	34.938
		Average Relative Error	17.997
MoReLab	14.046	11.564	17.672
	12.3953	11.761	5.117
	4.115	4.147	**0.783**
	2.616	2.584	**1.231**
	2.057	2.015	**2.061**
		Average Relative Error	**5.373**

**Table 3:** Results of comparing MoReLab against Metashape and 3-Sweep in terms of relative error in measurements on the second video (see Figure 11).

Method	Ground Truth (cm)	Measured Distance (cm)	Relative Error
Metashape	3.355	4.161	24.011
	3.216	3.109	**3.316**
	2.365	2.532	**7.073**
	2.251	2.626	16.688
	1.923	2.045	6.344
		Average Relative Error	11.486
3-Sweep	3.355	2.388	28.833
	3.216	2.868	10.817
	2.365	1.954	17.374
	2.251	1.905	15.359
	1.923	1.264	34.249
		Average Relative Error	21.326
MoReLab	3.355	4.083	**21.687**
	3.216	3.652	13.570
	2.365	2.594	9.695
	2.251	2.462	**9.401**
	1.923	1.926	**0.15**
		Average Relative Error	**10.902**

**Table 4:** Results of comparing MoReLab against Metashape and 3-Sweep in terms of relative error in measurements on the third video (see Figure 12).

Method	Ground Truth (cm)	Measured Distance (cm)	Relative Error
Metashape	35	39.837	13.82
	7	5.532	20.971
	6.9	7.254	5.13
	6.8	6.523	4.074
	6.7	6.396	4.537
		Average Relative Error	9.706
3-Sweep	35	36.913	**5.466**
	7	7.944	13.486
	6.9	7.251	**5.087**
	6.8	6.276	7.706
	6.7	7.532	12.418
		Average Relative Error	8.833
MoReLab	35	38.796	10.846
	7	7.817	**11.671**
	6.9	7.820	13.333
	6.8	6.858	**0.853**
	6.7	6.713	**0.194**
		Average Relative Error	**4.546**

**Table 5:** Results of comparing MoReLab against Metashape and 3-Sweep in terms of relative error in measurements on the fourth video (see Figure 13).

Method	Ground Truth (cm)	Measured Distance (cm)	Relative Error
Metashape	24	24.45	**1.873**
	17.5	15.959	8.843
	5	3.558	28.852
	4.2	3.528	15.974
	3.5	4.016	14.741
		Average Relative Error	14.057
3-Sweep	24	21.618	9.927
	17.5	12.287	29.817
	5	3.685	26.289
	4.2	5.228	24.461
	3.5	3.815	9.221
		Average Relative Error	19.943
MoReLab	24	21.51	10.375
	17.5	16.739	**4.349**
	5	4.592	**8.16**
	4.2	3.575	**14.881**
	3.5	3.621	**3.457**
		Average Relative Error	**8.244**

**Table 6:** Results of comparing MoReLab against Metashape and 3-Sweep in terms of relative error in measurements on the first video seen in Figure 14. The 1-measurement calibration table corresponding to this one is Table 2.

Method	Ground Truth (cm)	Measured Distance (cm)	Relative Error
Metashape	4.115	2.918	29.085
	2.616	2.213	15.412
	2.057	1.864	9.4
		Average Relative Error	17.966
3-Sweep	4.115	4.549	10.552
	2.616	3.077	17.613
	2.057	1.632	20.677
		Average Relative Error	16.281
MoReLab	4.115	4.497	**9.288**
	2.616	2.678	**2.362**
	2.057	2.073	**0.758**
		Average Relative Error	**4.136**

**Table 7:** Results of comparing MoReLab against Metashape and 3-Sweep in terms of relative error in measurements on the second video seen in Figure 15. The 1-measurement calibration table corresponding to this one is Table 3.

Method	Ground Truth (cm)	Measured Distance (cm)	Relative Error
Metashape	2.25	2.535	12.645
	1.923	2.07	**7.644**
	2.365	2.512	6.227
		Average Relative Error	8.839
3-Sweep	2.25	2.375	5.535
	1.923	1.554	19.189
	2.365	2.202	6.878
		Average Relative Error	10.534
MoReLab	2.25	2.272	**0.958**
	1.923	1.753	8.84
	2.365	2.431	**2.802**
		Average Relative Error	**4.20**

**Table 8:** Results of comparing MoReLab against Metashape and 3-Sweep in terms of relative error in measurements on the third video seen in Figure 16. The 1-measurement calibration table corresponding to this one is Table 4.

Method	Ground Truth (cm)	Measured Distance (cm)	Relative Error
Metashape	6.9	5.649	18.13
	6.8	6.482	4.676
	6.7	6.447	3.776
		Average Relative Error	8.861
3-Sweep	6.9	6.962	**0.899**
	6.8	7.940	16.765
	6.7	6.332	5.493
		Average Relative Error	7.719
MoReLab	6.9	7.41	7.391
	6.8	6.553	**3.632**
	6.7	6.776	**1.134**
		Average Relative Error	**4.052**

**Table 9:** Results of comparing MoReLab against Metashape and 3-Sweep in terms of relative error in measurements on the fourth video seen in Figure 17. The 1-measurement calibration table corresponding to this one is Table 5.

Method	Ground Truth (cm)	Measured Distance (cm)	Relative Error
Metashape	5	3.965	20.7
	4.2	3.164	24.667
	3.5	3.894	11.257
		Average Relative Error	18.875
3-Sweep	5	3.787	24.26
	4.2	4.991	18.833
	3.5	3.767	7.629
		Average Relative Error	16.907
MoReLab	5	4.585	**8.3**
	4.2	4.022	**4.238**
	3.5	3.547	**1.343**
		Average Relative Error	**4.627**

## Data Availability

The first dataset presented in this study is not available; but, the second dataset can be provided on request.

## Abbreviations

The following abbreviations are used in this manuscript:

SfM	Structure from Motion
MoReLab	Movie Reconstruction Laboratory
